# Relief for Medical Men

**Published:** 1871-12

**Authors:** 


### Relief for Medical Men.

We take great pleasure in calling attention to the following appeal in behalf of the medical men of Chicago, sufferers by the great fire:

CHICAGO, October 19, 1871.

At a meeting of physicians held on the 17th inst., at No. 798 Wabash avenue, of which Dr. N. S. Davis was made Chairman, and Dr. E. Andrews, Secretary; the announcement having been made that communications had been received from prominent Physicians of other cities, to the effect that contributions for the relief of the suffering members of the profession here, are now awaiting the order of responsible parties to receive and distribute them. Drs. Moses Gunn, E. Andrews and A. Fisher having been appointed a committee to recommend suitable persons for a permanent Relief Committee of five, nominated the following gentlemen: Drs. S. N. Davis, De Laskie Miller, Ernst Schmidt, T. D. Fitch and Walter Hay, which nominations being unanimously approved, the following resolutions were adopted:

*Resolved*, That the Committee just chosen is hereby authorized to receive all donations for the relief of the respectable Physicians, who are sufferers by the late fire, distribute the same at their discretion, and render a strict account, with vouchers, to any future meeting, which may be called by the Chairman, to consider the same.

*Resolved*, That this meeting tender the cordial and heartfelt thanks of the profession of this city to their brethren in other and distant cities, for the prompt and liberal offers of assistance to the many among us who have lost, by the late terrible fire, not only their homes, clothes, books and instruments but their practice, and pledge a just use of whatever is given.

Contributions may be forwarded at once by express, or draft on New York, to Walter Hay, M. D., Secretary Medical Relief Committee, No. 384 Michigan avenue.

Donations from publishing houses, instrument makers and physicians, of books, instruments, or apparatus, will be gratefully received; as many of our professional brethren have saved only their lives.

DE LASKIE MILLER, M. D.
No. 518 Wabash Avenue, Chairman.
N. S. DAVIS, M. D.,
No. 797 Wabash Avenue, Treasurer.
ERNST SCHMIDT, M. D.,
No. 387 State Street.
T. D. FITCH, M. D.,
No. 296 West Monroe Street.
WALTER HAY, M. D.,
No. 384 Michigan Avenue, Secretary.

} Committee.

